# Influence of the Mind over the Body

**Published:** 1882-12

**Authors:** 


### INFLUENCE OF THE MIND OVER THE BODY.

EVERY physician realizes the fact that a most important element in all diseases, is the mental condition of the patient. Although a complicated and wonderful machine, whose vital movements are involuntary, and whose mysteries we cannot comprehend, the body is the obedient servant of the will, and completely under its control.

Almost every one has had the opportunity of observing the influence of the mind in overcoming diseases, real and imaginary.

The opinion of "Dombey's" sister, that Mrs. Dombey would have lived if she had only "made an effort," we believe has been quoted, more as the expression of a generally recognized fact, than because of its oddity.

The determination to live in spite of a disease—that eminent physicians predicted would prove fatal in a few days—added more than twenty years to the life of a noted capitalist, and when he finally died of *old age*, it was said--perhaps facetiously--that each vital organ was impaired to an extent that would have long before destroyed the life of any *other* person. There are numerous and well authenticated cases of death through imagination alone, and quite as many instances of speedy recovery from imaginary diseases, upon proof that no disease existed. Dyspeptics are very apt to imagine that they have "heart disease." One of the most common effects of "worry" over troubles, real or imaginary, is a functional derangement of the secretions, which the patient generally imagines is "Bright's Disease." Some public speakers are never able to overcome their diffidence, and after preaching a sermon or delivering an address, invariably have an attack of "bowel trouble." So intimate is the relation between mind and body, that every emotion, whether of pleasure or of pain, makes an impression upon some vital organ, or betrays itself through various changes of the features that are as far beyond the control of the individual as are the movements of the planets.

